# An Improved Q-Learning-Based Sensor-Scheduling Algorithm for Multi-Target Tracking

**DOI:** 10.3390/s22186972

**Published:** 2022-09-15

**Authors:** Zhiyi Qu, Xue Zhao, Huihui Xu, Hongying Tang, Jiang Wang, Baoqing Li

**Affiliations:** 1 Science and Technology on Micro-System Laboratory, Shanghai Institute of Microsystem and Information Technology, Chinese Academy of Sciences, Shanghai 201800, China; 2 University of Chinese Academy of Sciences, Beijing 100049, China

**Keywords:** wireless sensor networks, multi-target tracking, sensor scheduling, target priority, task allocation, tracking accuracy, energy efficiency

## Abstract

Target tracking is an essential issue in wireless sensor networks (WSNs). Compared with single-target tracking, how to guarantee the performance of multi-target tracking is more challenging because the system needs to balance the tracking resource for each target according to different target properties and network status. However, the balance of tracking task allocation is rarely considered in those prior sensor-scheduling algorithms, which may result in the degradation of tracking accuracy for some targets and additional system energy consumption. To address this issue, we propose in this paper an improved Q-learning-based sensor-scheduling algorithm for multi-target tracking (MTT-SS). First, we devise an entropy weight method (EWM)-based strategy to evaluate the priority of targets being tracked according to target properties and network status. Moreover, we develop a Q-learning-based task allocation mechanism to obtain a balanced resource scheduling result in multi-target-tracking scenarios. Simulation results demonstrate that our proposed algorithm can obtain a significant enhancement in terms of tracking accuracy and energy efficiency compared with the existing sensor-scheduling algorithms.

## 1. Introduction

Currently, wireless sensor networks (WSNs) have attracted worldwide attention due to opening new vistas for a wide range of application domains [1,2,3]. As one of the attractive applications in WSNs, target tracking has made contributions in many fields such as frontier security, illegal vehicle tracking, space exploration, and pasture protection [4,5]. In such application scenarios, the tracking network can be constructed from multiple energy-constrained sensor nodes. During the tracking process, some sensors are required to be activated to locate mobile targets, whereas other sensors can switch into a dormant state to reduce energy consumption.

Most of the existing target-tracking algorithms focus on single-target tracking or tracking of multiple targets with identical properties. However, in practical applications, the system may need to track different types of targets, and the network status where targets are located may also be different. Compared with single-target-tracking scenarios, how to schedule sensors in such complex multi-target-tracking scenarios is a more challenging research work [6]. Therefore, it is necessary to study a novel sensor-scheduling algorithm for multi-target tracking.

## 2. Related Work

Most of the recent research in tracking-sensor networks focuses on improving the performance of sensors performing collaborative tracking tasks. Generally, the existing works are designed for single-target tracking or tracking of multiple homogeneous targets.

In [7], a dynamic chain-based collaboration (DCBC) method was proposed for efficient target tracking and data gathering. In DCBC, sensors form a dynamic tracking chain around the target, which can adjust its structure as the target moves. The sensing data collected by tracking nodes is aggregated locally at each time step. In this way, DCBC can reduce the energy consumption of target tracking and prolong the network lifetime.

In [8], an energy-aware task-scheduling approach (TOTD) was proposed to achieve a trade-off between energy efficiency and tracking performance. The authors utilized a true online reinforcement learning framework to obtain the optimal task scheduling scheme for sensors to track targets. Simulation results showed that TOTD outperforms other methods for target-tracking applications.

Ramluckun et al. proposed a chain-cluster based routing protocol in [9], in which the combination of clustering and ant colony optimization (ACO) was used to reduce transmission delay and obtain the optimal routing path. In the simulation, this protocol was proved superior in residual energy, latency, and load balancing, making it suitable for target tracking.

To manage the problem of selecting the optimal sensors for target tracking, Anvaripour et al. proposed a reliable sensor selection approach in [10]. They proposed an updated unscented Kalman filter (U2KF) to improve the efficiency of target tracking by sensor selection. Moreover, multi-objective optimization was utilized to select a sensor set without knowing the number of sensors needed to be selected. Sigma points probability (SP) and target trajectory (TT) approaches were utilized to eliminate the effects of noise while selecting. The superiority of Anvaripour et al.’s method in effectiveness and utility was proven in extensive experiments.

A dynamic cluster establishment scheme and a distributed consensus-based adaptive Kalman estimation method were proposed by Zhang et al. [11] to reduce energy consumption and improve estimation accuracy in target tracking.

In [12], a dynamic cluster member scheduling (DCMS) algorithm was proposed by Wu et al. to achieve the trade-off between energy efficiency and tracking performance; this method constructed a reward function by exploiting the weighted value. Then, a point-based online value iteration algorithm was designed to schedule sensors to collaboratively track targets. In simulations, DCMS was shown to improve tracking accuracy and extend network lifetime.

Combined with the particle swarm optimization (PSO) algorithm, Pang and Shan [13] proposed an improved bee colony algorithm based on a double-roulette scheme to obtain the optimal sensor-scheduling scheme. Additionally, to reduce the target-missing risk and the sensor radiation interception risk, they created the sensor-scheduling objective function which takes risk factors into account.

The authors in [14] proposed a dynamic-clustering protocol (EEDC) for target tracking and continuous event monitoring. In EEDC, the overlapping cluster structure was dynamically reconstructed to adapt to the changing location of targets, and candidate cluster heads were selected according to rough fuzzy C-means (RFCM) and genetic algorithm (GA). EEDC can significantly reduce system energy consumption as compared to other clustering protocols.

The authors in [15] proposed a hierarchical-tracking structure based on edge intelligence (EI) technology. The structure can integrate the computing resources of sensors and edge servers to provide efficient computing for real-time tracking. Moreover, the authors designed a long-term dynamic resource allocation algorithm to obtain the optimal sensor-scheduling scheme. Simulation results demonstrated that the proposed scheduling method can enhance tracking accuracy and reduce energy consumption.

In [16], Liu et al. proposed a message-pruning tree with shortcuts, which can reduce the total cost of updating the database and querying targets compared with the message-pruning tree.

A tree-based multi-cast routing protocol was proposed in [17]. Based on residual energy and minimum hop count paths, this method can enhance multiple service quality parameters, including energy consumption, end-to-end delay, and packet delivery rate, by updating the multi-cast tree structure.

Overall, most of the existing sensor-scheduling algorithms focus on improving the performance of tracking networks by studying how to organize and schedule nodes to perform tracking tasks in a collaborative manner. Their goal is to select an optimal set of tracking nodes based on metrics such as sensor energy and distance to the target. However, in multi-target-tracking scenarios, it is insufficient to consider these metrics in the scheduling process. Owing to the lack of consideration for the difference in target properties and the difference in network status where targets are located, the existing sensor-scheduling algorithms may face the problem of unbalanced allocation of tracking resources for the targets when managing multi-target tracking.

**Figure 1:**
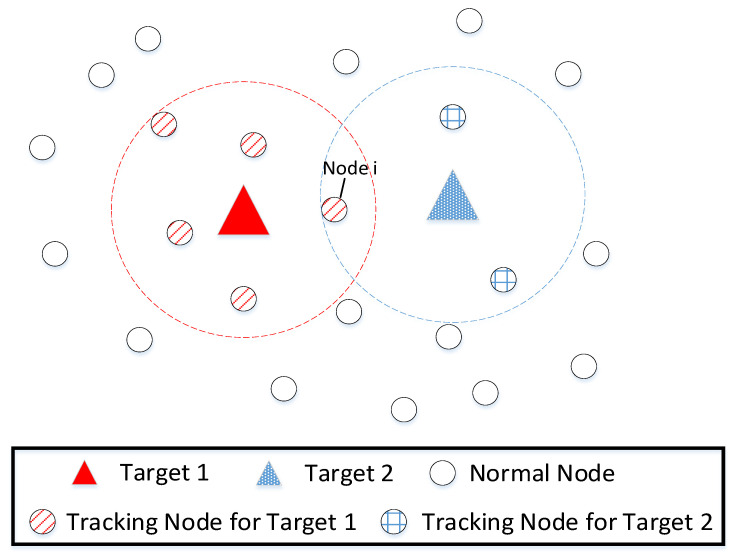
Traditional nearest-neighbor-scheduling method.

For example, Figure 1 shows the scheduling results of sensors using the classical nearest-neighbor method. It can be seen that, according to the nearest-neighbor principle, node i should work as a tracking node for target 1. However, target 2 has only two tracking nodes, and the system lacks sufficient localization information for target 2, so the tracking accuracy is seriously affected. Moreover, the two tracking nodes of target 2 have to undertake heavy tasks, which may cause unbalanced energy consumption. In this case, target 2 should be given higher priority than target 1 when scheduling the tracking task for node i. Besides the nearest-neighbor method, other algorithms that do not consider the balance of resource allocation in scheduling will encounter similar problems.

It can be seen from the example in Figure 1 that once such an unbalanced task allocation occurs in the sensor-scheduling process, it leads to the degradation of the overall system performance, such as tracking accuracy and energy efficiency. Therefore, to address this issue, we propose a sensor-scheduling algorithm with consideration of the balance of tracking-task allocation for multi-target tracking (MTT-SS). The contributions of this paper are summarized as follows:

Taking advantage of the entropy-weight method (EWM), MTT-SS calculates the aggregated weight for each target according to four decision criteria, including target type, distance to the cluster center, node density, and energy level. After that, linear weighting is conducted to obtain the overall priority function to evaluate target priority. Prioritizing targets can help sensors select appropriate tasks to execute based on the current status of targets and the network.

To obtain a balanced resource scheduling result, a Q-learning-based task allocation mechanism is developed, in which nodes are mapped as intelligent agents. Each agent selects the appropriate task to execute by using an improved exploration–exploitation policy. At each time step, agents update their Q value according to the environment state and the delayed reward.

Extensive experiments and analyses for different solutions are presented in the simulation. Simulation results indicate that MTT-SS significantly outperforms other existing sensor-scheduling algorithms in terms of tracing accuracy and energy efficiency.

Following the related work given in Section 2, Section 3 describes the network model and the energy consumption model. The proposed sensor-scheduling algorithm is presented in Section 4. Section 5 provides the simulation results. Finally, Section 6 concludes this paper.

## 3. System Model

We consider a homogeneous network for multi-target tracking and make assumptions as follows [7,18]:

1. In a two-dimensional monitoring area, sensor nodes are randomly deployed. The sink node, which is responsible for processing sensing data and connecting to the external network, is placed at the center of the surveillance area. All these sensor nodes are stationary after deployment.
2. Equipped with GPS, each node can obtain its location information. All the nodes are initially charged with identical energy and the sink node has no energy constraints.
3. The communication range of each node $r_{c}$ is set at twice its sensing range $r_{s}$ so that all the nodes that sense the target can communicate with each other.
4. The wireless channel is completely symmetrical, hence the energy consumption of transmitting a package along the back and forth route is equivalent.

To calculate the energy consumption of nodes during transmission and reception, we adopt the first-order energy model [19] in which the free space model and multi-path fading channel model are used to calculate path loss. If the transmitter–receiver distance is less than or equal to the threshold $d_{0}$, then the free space model is selected; otherwise, multi-path is selected. The energy consumption for transmitting and receiving l-bit messages over d is given by

(1) $$E_{Tx}(l,d)=\left\{ \begin{array}{lll} l\cdot E_{elec}+l\cdot \varepsilon_{fs}\cdot d^{2}, & if & d<d_{0}\\ l\cdot E_{elec}+l\cdot \varepsilon_{mp}\cdot d^{4}, & if & d\geq d_{0} \end{array}\right.$$

(2) $$E_{Rx}=l\times E_{elec},$$

where $E_{elec}$ is the energy consumption due to circuit processing before the transmission of a data packet and after the reception of a data packet, while the amplifier energy, given by $\varepsilon_{fs}\times d^{2}$ and $\varepsilon_{mp}\times d^{4},$ depends on the distance d and the specified bit-error rate; $d_{0}$ is the distance threshold, which can be given by

(3) $$d_{0}=\sqrt{\varepsilon_{fs}/\varepsilon_{mp}}.$$

Typical values for the parameters $\varepsilon_{fs}$ and $\varepsilon_{mp}$ are given in Table 1.

## 4. Detailed Description of the Proposed Algorithm

The proposed sensor-scheduling algorithm for multi-target tracking is composed of three mechanisms: dynamic clustering, target prioritizing, and task allocation. In what follows, we elaborate on each of the three component mechanisms.

### 4.1. Dynamic Clustering

Cluster, as the most commonly used network structure, is widely used to reduce energy consumption and prolong network lifetime. In the clustering structure, sensors are classified into the cluster head (CH) and cluster members (CMs). CH has stronger abilities than CMs; it can collect the data from its CMs, perform data fusion to remove transmission redundancy, and forward the data to the sink node. Additionally, CH can provide necessary target information for its member nodes. Therefore, in the first step of our scheduling algorithm, sensors are organized into clusters to facilitate collaborative data processing in the network. The traditional clustering method is that the sink node selects several sensors with strong capability as the CHs, and determines their subordinate CMs. However, this approach is not suitable in target-tracking scenarios, since most of the sensors in the network do not need to participate in the tracking tasks. Therefore, we employ a delayed broadcasting mechanism in which CH voluntarily recruits sensors to form a dynamic cluster. In this way, we can reduce the number of nodes forming clusters in the network, minimizing unnecessary energy consumption. First, the sensors that can sense the target will set a back-off timer and broadcast its competing message when the timer expires. If by the time the back-off timer expires, the sensor receives a competing message from other sensors, it cancels the timer. Specifically, the back-off time, $T_{back-off}$, for which sensor i uses is defined as [18,20]:

(4) $$T_{back-off}(i)=T_{\min}+(T_{\max}-T_{\min})*(\mu \frac{\bar{d_{i}}}{e_{re}(i)})+T_{rand},$$

where $T_{\min}$ and $T_{\max}$ denote the minimum and maximum back-off timer values; $\bar{d_{i}}$ is the average distance from node to targets; $e_{re}(i)$ is the residual energy of sensor; and μ is a parameter which can nondimensionalize the second term in Equation (4). μ is given by

(5) $$\mu =e_{0}/r_{s},$$

where $e_{0}$ is the initial energy of the sensor and $r_{s}$ is the node sensing range, which denotes the maximum distance from the node to targets. Moreover, the third term in Equation (4) is a random part.

According to Equation (4), the delay determined by the back-off timer value can ensure that the sensor with a shorter average distance to targets and more residual energy will broadcast the competing message earlier, and thus have a higher chance of serving as the CH.

### 4.2. Target Prioritizing

In this subsection, to manage the problem of unbalanced task allocation, we first prioritize the targets according to target properties and network status. We introduce four indicators as follows to evaluate the priority of targets:

***(1)***  **Target Type** 

For different types of targets, the system pays different attention to them. For example, in military applications, systems have different importance for monitoring soldiers and armored vehicles because of their differences in speed and combat effectiveness. Therefore, it is necessary to distinguish the type of target and give an evaluation metric about its importance, which can be used as a reference for sensor scheduling. We use η to measure the importance of the targets. The value of η for different targets is evaluated and normalized according to specific applications requirements, so we have η∈(0,1).

***(2)***  **Distance to cluster center** 

Distance to cluster center D is obtained by calculating the Euclidean distance between the target and its corresponding cluster center. When this target moves to the boundary of its cluster, it may activate new clusters. In this case, the system needs a precise estimate of the target position to prevent target loss caused by activating the wrong clusters.

***(3)***  **Node Density** 

Node density ρ indicates the distribution density of the nodes around the target. Owing to the fact that sensors are randomly deployed, there are areas where nodes are sparsely distributed in the network. When a target moves to this area, the system should give priority to it to ensure tracking reliability.

***(4)***  **Energy Level** 

Energy level E indicates the residual energy of the nodes around the target. As the tracking system works, there may be nodes that take on the tracking task multiple times, resulting in greater energy consumption than others. To ensure the reliability of the tracking process, the target, whose surrounding nodes have low residual energy, should be set as a high tracking priority.

To determine the weights of different indicators, we use EWM to calculate the comprehensive score for target priority. EWM is an objective weighting method based on the degree of data dispersion for each indicator [21]. EWM first uses the information entropy principle to calculate the entropy value of each indicator, and then corrects the entropy weight according to the data distribution of each indicator, so as to obtain the objective index weight. Compared with subjective methods such as analytic hierarchy process (AHP), EWM has better accuracy and adaptability. EWM includes the following steps:

First, we normalize all the indicators by using the extreme-value processing method. When the indicator has positive effects, we have:

(6) $$Y_{ij}=\frac{X_{ij}-X_{i,\min}}{X_{i,\max}-X_{i,\min}}.$$

When the indicator has negative effects, we have:

(7) $$Y_{ij}=\frac{X_{i,\max}-X_{ij}}{X_{i,\max}-X_{i,\min}}.$$

where $Y_{ij}$ is the normalized value; $X_{ij}$ is the value of the $j^{th}$ indicator of the $i^{th}$ target; and $X_{i,\max}$ and $X_{i,\min}$ are the maximum and minimum values of the corresponding indicator, respectively. After normalization, the entropy $E_{j}$ of the $j^{th}$ indicator is given by:

(8) $$E_{j}=-k\sum_{i=1}^{n}f_{ij}\ln f_{ij},\;j=1,2,\ldots ,m$$

(9) k=1/lnn,

where m is the number of indicators; n is the number of targets; $f_{ij}=Y_{ij}/\sum_{i=l}^{n}Y_{ij}$ and assuming that when $f_{ij}=0,f_{ij}\ln f_{ij}=0$. The function of k in Equation (9) is to make the entropy value fall within (0, 1). We then define the information utility value, which can measure the amount of useful information contained in the indicator. It can be learned from Equation (8) that, when values of indicators of all samples are equal, E obtains a maximum value, which means that data do not change, that is, there is little information. Thus, we reverse it to calculate the information utility value $H_{j}$ of the $j^{th}$ indicator:

(10) $$H_{j}=1-E_{j}.$$

Then, we determine the weight of each indicator by normalizing the information utility value:

(11) $$\omega_{j}=H_{j}/\sum_{j=1}^{m}H_{j},$$

where $\omega_{j}$ is the weight of the $j^{th}$ indicator. Finally, the comprehensive evaluation of target priority P is obtained:

(12) $$P(o_{i})=\omega_{1}\eta +\omega_{2}D+\omega_{3}\rho +\omega_{4}E,$$

where $o_{j}$ denotes the $i^{th}$ target.

When the value difference between the evaluating targets of the same indicator is large and the entropy is small, it indicates that this indicator provides more useful information than other indicators. Accordingly, the weight value of this indicator is higher. By carrying out EWM, we can avoid errors caused by the prejudice of decision-makers and thus have high accuracy in weight identification.

### 4.3. Task Allocation

To allocate tasks to sensors reasonably, we propose, with the use of target priority information, a Q-learning-based multi-target-tracking task allocation mechanism. In this subsection, we first map the sensor network to the Q-learning model. Then, we present the self-driven process of the mechanism and detail the setting of the reward function, which can evaluate the performance of the strategy at each step in Q-learning. Finally, we improve the exploration–exploitation policy according to the principle of balancing task allocation.

#### 4.3.1. Reinforcement Learning and Q-Learning Model

Reinforcement learning (RL) mainly discusses how agents can maximize their rewards in the environment. RL consists of three parts: environment, action, and reward. After obtaining the status from the environment, the agent uses this status to output an action. After the action is executed, the environment outputs the next status and the delayed reward. Delayed reward is a scalar feedback signal sent by the environment to an agent, which can indicate how the agent performs when adopting a strategy at a certain step. We focus on RL for two reasons. First, the RL model can adapt well to the complexity and uncertainty of multi-target-tracking scenarios. Second, RL is free from the artificial label of supervised learning and explores the optimal strategies by agents themselves, which may be able to achieve performance beyond human capabilities.

**Figure 2:**
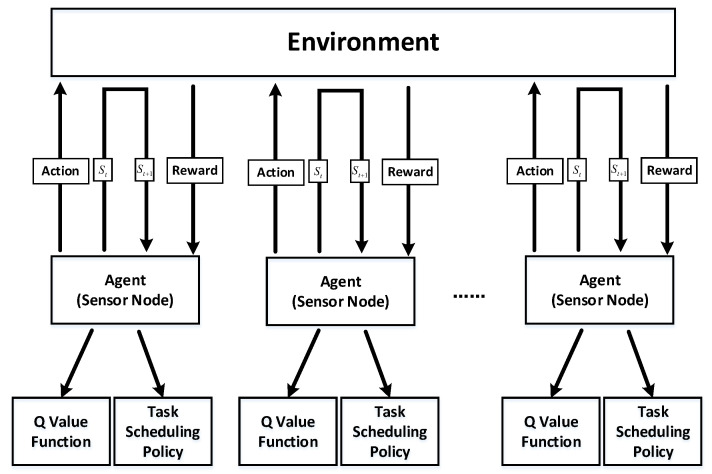
Proposed task-scheduling mechanism based on Q-learning.

Q-learning is a value-based model-free RL algorithm, in which agents do not need to estimate state transitions or formulate explicit policies, but maintain a value table. The agent selects the most valuable action through this table. That is, the Q-learning model can provide what the optimal action is in the current state, but it will not provide which next state the agent will move to. Therefore, Q-learning is suitable for multi-target-tracking scenarios where the environmental state is difficult to estimate and the action set is discrete and limited. In contrast, Q-learning is more generalizable than the model-based method, which requires modeling a virtual environment. Since our training process is describable, and the state of the agent is discrete and observable, we do not need the state transition function, and we can obtain good model performance by training samples. In our model based on Q-learning, sensor nodes in the network can be mapped as agents. Each agent observes the current state of the environment and performs an action according to the exploration–exploitation policy. After that, the environment moves to the next state and produces a reward to strengthen or weaken the trend of the agent to choose this action. The system chooses actions for the sensors based on the value table and based on the principle of maximizing the agent’s value function. Our proposed Q-learning-based task-scheduling mechanism is shown in Figure 2.

**Figure 3:**
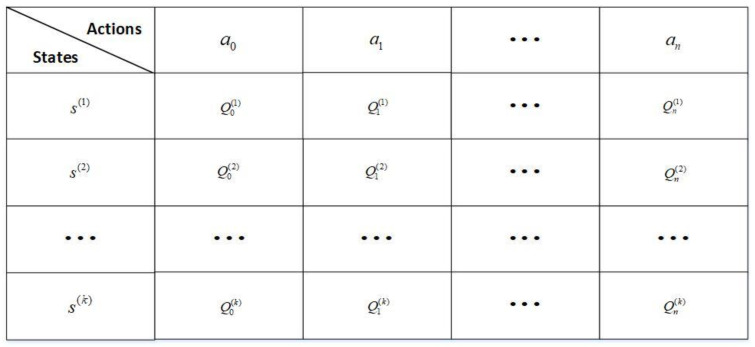
Q table.

Our goal is to maximize the cumulative reward and obtain the optimal scheduling decision. The proposed model can be described as follows:

Each sensor node acts as an intelligent agent that can make its own decision and the tracking status of targets in the network represents the environment in our model. Interaction between agents and the environment is achieved by executing actions and receiving a reward function. According to the reward of each action taken by the agent in various states during training, we summarize a Q table. As shown in Figure 3, the ordinate of the Q table is the state, the abscissa is the action, and the Q value is obtained from the Q value of the previous state and the estimated future reward, which is given by Equation (13). Every time the agent is in a certain state, the next action is to retrieve from the table the future reward with the highest value. If there are multiple identical values, one of them is randomly selected. Repeat until finished. This operation is repeated until the final state is reached.

***(1)***  **Action Space** 

The set of actions $𝒜=\{a_{0},a_{1},\ldots ,a_{n}\}$ is a set that collects all the targets that the sensors in the cluster can sense, and n denotes the target number. When the sensor executes action $a_{i}$, it means it selects to track the i-th target. In particular, executing action $a_{0}$ indicates that the sensor does not sense any target and goes to sleep.

***(2)***  **State Space** 

The environment state that the agent is in at each time step constitutes the state space S, $S=\{s^{1},s^{2},\ldots ,s^{k}\}$. In our model, $s^{\left(l\right)}$ denotes the targets that do not meet the tracking quality requirements in the surveillance area of the sensors at the l-th time step.

***(3)***  **Reward** 

The reward obtained by the agent executing the j-th action at the l-th time step is expressed as $R_{j}^{\left(l\right)}$. The specific numerical setting of the reward function is given by Equation (14). The reward provides the evaluation of the actions executed by agents in a certain environment state. The reward function in our proposed model is designed for the trade-off between energy efficiency and tracking quality of the sensor in a multi-target-tracking scenario.

#### 4.3.2. Learning Process

In the learning process, each sensor needs to maintain a *Q* table, as shown in Figure 3. Initially, all entries in the *Q* table are zero. Sensors select an action from the action space to execute at each time step. The environment then moves to the next state based on the action taken by the agent. The update of the *Q* values is calculated by the following rules:

(13) $$Q^{\left(t\right)}=(1-\beta )Q^{\left(t\right)}+\beta [R^{\left(t\right)}+\gamma \underset{a}{\max}(Q_{a}^{\left(t+1\right)}-Q^{\left(t\right)})].$$

where β∈[0,1] is the learning rate that controls the learning speed; γ is the discount factor, which we set to 0.8 to consider the expected reward in the near future. The reward function ℛ for the agent is defined as

(14) $$ℛ(a_{i})=\left\{ \begin{array}{ll} (n_{0}-n_{i})(\lambda \frac{e_{res}}{e_{init}}+(1-\lambda )\frac{r_{s}-d_{i}}{r_{s}}),\; & i\neq 0\\ (1-m_{not})\frac{e_{init}-e_{res}}{e_{init}},\; & i=0 \end{array}\right.,$$

where λ∈[0,1] is a weight coefficient; $e_{res}$ is the residual energy; $e_{init}$ is the initial energy; $r_{s}$ is sensing range; $d_{i}$ is the distance to the i-th target; $n_{0}$ is the number of nodes required to track a single target; $n_{i}$ is the number of nodes that choose to track the i-th target currently; and $m_{not}$ is the number of targets that are not allocated enough tracking nodes.

According to Equation (14), when i≠0, the node chooses to track the i-th target. In this manner, if $n_{i}<n_{0}$, then the node is qualified obtains a positive reward. Otherwise, the node is a redundant tracking node and receives no reward or one that is negative. The reward function consists of two parts: energy and distance; more rewards are given to nodes with more residual energy and closer to the target.

On the other hand, when i=0, the node chooses to go to sleep. In this manner, if $m_{not}=0$, all targets meet tracking requirements, it is reasonable for the node to choose to sleep, and a positive reward can be obtained. Otherwise, if $m_{not}\neq 0$, there are targets that do not meet tracking requirements. In this case, the node should assist in the tracking task, so choosing to sleep receives zero or negative rewards. Since the node chooses to sleep, the reward function no longer includes tracking-related indicators. More rewards will be given to nodes with low residual energy.

The agent will choose the most rewarding action in the *Q* table at each time step or randomly select an action to execute according to the exploration–exploitation strategy. This process repeats until the maximum number of iterations is reached. By this iterative calculation, each agent learns the *Q* value of performing different actions in different environmental states, and then updates the *Q* table. After the iteration of the *Q* model is completed, we simply need to look up the final *Q* table to obtain the agent’s optimal policy $\pi^{*}$, that is, the optimal scheduling strategy for sensor resource allocation in the multi-target-tracking scenario. $\pi^{*}$ can be expressed as

(15) $$\pi^{*}==\arg\underset{a\in A}{\max}Q(S,a).$$

#### 4.3.3. Exploration–Exploitation Policy

The exploration–exploitation policy has a direct effect on learning efficiency in Q-learning [22]. Most of the existing research uses the greedy policy, and the exploration probability ρ is given by

(16) $$\rho =\min(\rho_{\max},\rho_{\min}+k*(S_{\max}-S)/S_{\max}),$$

where $\rho_{\max}$ and $\rho_{\min}$ are the upper and lower boundaries for the exploration factor, respectively; k∈[0,1] is a fixed constant; $S_{\max}$ is the maximum number of states; and S is the current number of states already known. At each time step, random action is selected with the exploration probability ρ and the optimal action is selected with the exploitation probability 1−ρ. However, this strategy cannot provide meaningful guidance for the agent to choose actions based on the real-time allocation of sensor resources in the network. To manage this issue, we propose, with the use of target priority information, a novel exploration probability ρ:

(17) $$\rho =\min(\rho_{\max},\rho_{\min}+(P_{\max}-P_{\min})/P_{\max}),$$

where $P_{\max}$ and $P_{\min}$ are the maximum and minimum values of the target priority. Note that if we obtain a more reasonable task allocation, then different targets obtain more balanced priorities in real-time. In the early learning process, the tasks are unevenly allocated, and the maximum differences in target priorities are larger, so the agent is encouraged to perform more exploratory actions. With the iterative learning process, the resource allocation scheme learned by the agent gradually becomes more balanced, thus ρ should be decreased to take more greedy actions to complete the convergence of the final Q-table.

The pseudocode of the proposed multi-target-tracking task allocation mechanism is shown in Algorithm 1.
**Algorithm 1.** The Q-learning based task allocation mechanism.**Input:** sensor nodes, n targets, state space S, action space A, Q table**Output:** The optimal policy for sensor scheduling1: **Parameter Initialization:** maximum number of iterations $t_{\max}$, learning rate β, discount factor γ2: Initial Q value in Q table is 0 for each node3: **repeat**4: **for** each node before $t<t_{\max}$
**do**
5: Evaluate the environment state $s^{\left(t\right)}$ at time stept6: Select an exploitation or exploration action according to Equation (17)7: **if** random(0, 1) < ρ
**then**8: Exploration: node choose a random action a, a∈A9: **else**10: Exploitation: node choose the action $a=\arg\max_{a\in A}Q^{\left(t\right)}(s^{\left(t\right)},a)$11: **end if**12: The corresponding delayed reward is calculated based on Equation (14)13 Observe the next state $S_{t+1}$ and update Q value as Equation (13):14: $Q^{\left(t\right)}=(1-\beta )Q^{\left(t\right)}+\beta [R^{\left(t\right)}+\gamma \underset{a}{\max}(Q_{a}^{\left(t+1\right)}-Q^{\left(t\right)})]$.15: end for16: The optimal policy for sensor scheduling $\pi^{*}$ can be obtained by:17: $\pi^{*}=\arg\max Q(S,A)$


**Figure 4:**
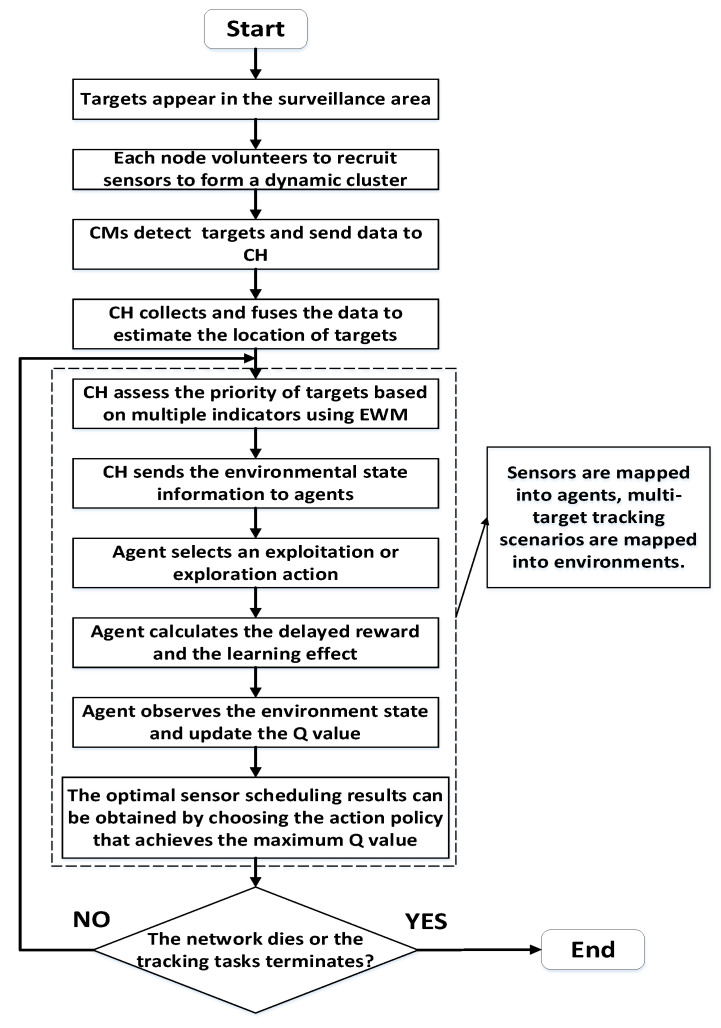
Flow chart of the proposed MTT-SS method.

In general, the operation process of our proposed improved Q-learning-based sensor-scheduling algorithm is as follows: Firstly, in subsection A, based on distance and energy information, sensors use a delay-based broadcast mechanism to select CH. CH can provide sensor nodes, i.e., intelligent agents, with the environment information and computation of rewards necessary for the Q-learning model. In subsection B, we propose four indicators according to target property and network status, and use EWM to calculate the tracking priority of targets. In Section 4.3, we detail the process of utilizing Q-learning models for task allocation. The reward function and the exploration–exploitation policy are designed to reasonably schedule nodes, thus achieving a balanced allocation of tracking tasks for targets. The overall flow chart of the proposed sensor-scheduling algorithm MTT-SS is shown in Figure 4.

## 5. Simulation Results

In this section, we make extensive experiments for our proposed algorithm to compare the performance with that of DPT [23], DIRL [24], TOTD [8], and DCBC [7]. Among these algorithms, DPT, which is a traditional sensor-scheduling algorithm, uses a cluster-based tracking framework and recovery mechanism to advance efficiency and robustness for target tracking. Both DIRL and TOTD are the reinforcement-learning-based algorithms proposed for tracking task management. In DIRL, sensors learn the utility of performing different tasks using local information to optimize global energy efficiency without losing significant accuracy. TOTD, which exploits a true online reinforcement learning algorithm, is an energy-aware task-scheduling algorithm aiming to achieve a better trade-off between tracking quality and energy consumption. By using a dynamic tracking chain, DCBC can improve the data collection quality and prolong the network lifetime. In the experiments, we evaluate these algorithms using a multi-target-tracking scenario implemented in the MATLAB R2016a simulation environment.

### 5.1. Simulation Setup

A total of 200 sensors are randomly deployed in a two-dimensional field ($100\times 100\;m^{2}$) and the sink is located at (50, 50). The sensing range and communication range of each sensor are identically fixed to 20m and 40m. The size of the control packet and data packet is 8 bytes and 256 bytes. The simulation lasted for 1000 time steps. The parameters that appeared in the simulations are listed in Table 1.

We simulate a scenario where multiple targets move based on a Gauss–Markov mobility model [25] in the surveillance area. Initially, a random speed and direction are assigned to each target. At each time step t, the movement parameters of targets are updated by

(18) $$v_{t}=\eta v_{t-1}+(1-\eta^{2})\bar{v}+\sqrt{1-\eta^{2}}v_{t-1}^{G},$$

(19) $$\theta_{t}=\eta \theta_{t-1}+(1-\eta )\bar{\theta }+\sqrt{1-\eta^{2}}\theta_{t-1}^{G},$$

where $v_{t}$ and $\theta_{t}$ are the current speed and direction of the target at time t; $\bar{v}$ and $\bar{\theta }$ are constants denoting the mean value of speed and direction; $v_{t-1}^{G}$ and $\theta_{t-1}^{G}$ are random variables from a Gaussian distribution; and η∈(0,1) is a parameter that is used to vary the randomness of target motion. Therefore, the position of the target is calculated as follows:

(20) $$\left\{ \begin{array}{l} x_{t}=x_{t-1}+v_{t-1}\cos(\theta_{t-1})\\ y_{t}=y_{t-1}+v_{t-1}\sin(\theta_{t-1}) \end{array}\right..$$

The localization method we use to calculate the target position is the weighted centroid localization (WCL) [26]. At each time step t, the estimated target position $\left(x_{t}^{est},y_{t}^{est}\right)$ is calculated based on the distance-related weights of N tracking nodes around it:

(21) $$\left\{ \begin{array}{l} x_{t}^{est}=\sum_{u=1}^{N}d_{u}^{-\xi }*x_{u}/\sum_{u=1}^{N}d_{u}^{-\xi }\\ y_{t}^{est}=\sum_{u=1}^{N}d_{u}^{-\xi }*y_{u}/\sum_{u=1}^{N}d_{u}^{-\xi } \end{array}\right.$$

where $d_{u}$ is the distance between the target and sensor u, and exponent ξ determines the weight of the contribution of each sensor. According to Equation (21), the closer the sensor is to the target, the more it contributes to the calculation of the target position.

### 5.2. Performance Analysis

We use two indicators to evaluate the performance of the multi-target-tracking sensor-scheduling algorithms, including tracking accuracy and energy efficiency.

In the first set of simulations, we evaluate the tracking accuracy of scheduling algorithms by comparing localization errors and the standard deviation of localization errors. Localization error is the average deviation (in meters) of the estimated location from the exact location of the targets. Based on Equations (20) and (21), average localization error can be calculated by

(22) $$\bar{error}=\frac{1}{n}\sum_{i=1}^{n}\sqrt{{\left(x_{t}^{est}-x_{i}\right)}^{2}+{\left(y_{t}^{est}-y_{i}\right)}^{2}},$$

where n is the number of targets in the surveillance area. Moreover, we use the standard deviation of localization errors σ for different targets to evaluate the balance of allocation of tracking resources, σ can be calculated by

(23) $$\sigma =\sqrt{\frac{1}{n}\sum_{i=1}^{n}{\left(error_{i}-\bar{error}\right)}^{2}},$$

where $error_{i}$ is the localization error of the i-th target. The smaller the value of σ, the smaller the difference in the tracking accuracy for each target, which means that the allocation of tracking tasks by the scheduling algorithm is more balanced.

**Figure 5:**
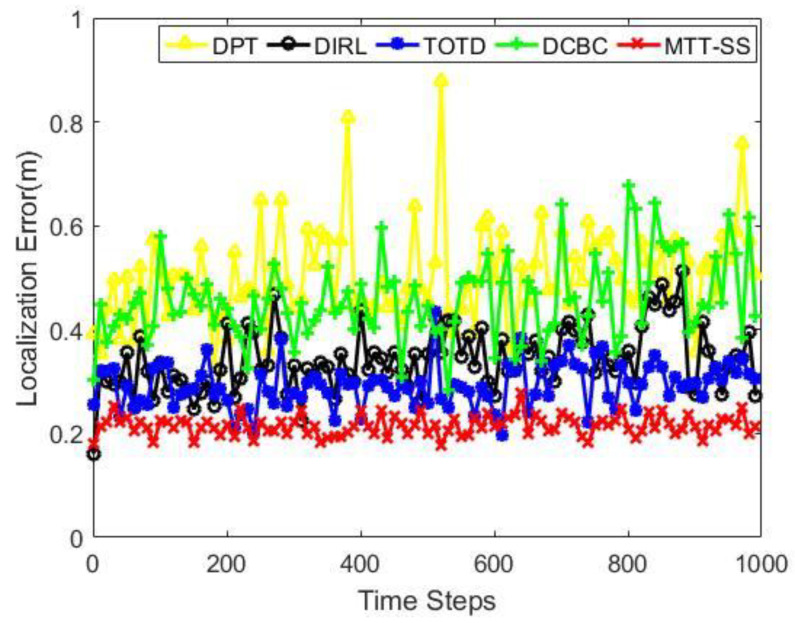
Localization error.

Figure 5 presents the comparison results of the localization errors in the case of 10 targets moving in the surveillance area. MTT-SS achieves lower localization errors than DPT, DIRL, TOTD, and DCBC, which are approximately 48%, 36%, 27%, and 42%, respectively. Additionally, Table 2 gives the standard deviation of localization errors for targets. Combining both results, it can be seen that MTT-SS outperforms DPT, DIRL, TOTD, and DCBC in terms of achieved tracking accuracy. Both DPT and DCBC perform poorly in terms of localization errors and standard deviation of localization errors. The reason is that they do not consider the balance of tracking resources in the scheduling process, which results in unreasonable task allocation. Moreover, DIRL and TOTD solve the multi-target-tracking problem based on the extent RL framework, but no tracking resource balance parameter is considered in the reward function. In contrast, MTT-SS first prioritizes targets with target attributes and network status. Using this information, the sensors can select higher priority targets for tracking under the same conditions. The setting of the reward function makes the sensors consider whether the resources required by the target meet the requirements to select the target to obtain more rewards. This method can balance the allocation of sensor resources among targets, and large tracking errors for individual targets rarely occur. This is why MTT-SS performs better than other methods in terms of tracking errors and the deviation of errors.

**Figure 6:**
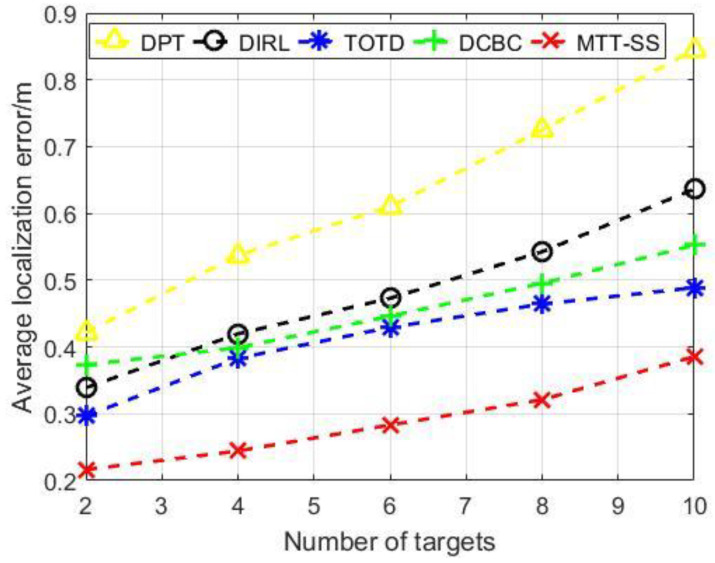
Average localization error vs. number of targets.

To examine the influence of target number on system performance, we gradually increase the number of targets while other parameters remain unchanged. As shown in Figure 6, the average localization error, which is calculated by averaging the localization errors of all targets for one simulation run, is proportional to the number of targets in the surveillance area. As the number of targets increases, it becomes more difficult for the system to ensure a balanced allocation of sensor resources among targets, which leads to insufficient sensing resources for some targets. This is the reason why the average tracking errors increase with the number of targets. Thus, it is important to design a proper allocation mechanism to allocate sensor resources to different targets. In DPT and DIRL, sensors randomly choose to perform tracking tasks at each time step. Furthermore, sensors in TOTD and DCBC perform a set of tracking tasks according to the distance minimization rule. It can be seen from Figure 6 that neither the traditional random allocation mechanism nor the minimum distance rule can achieve a smaller localization error in the tracking process because their rules do not consider the priority of tracking resource allocation or set a reasonable reward for the sensors. In contrast, MTT-SS first uses a target-prioritizing strategy to determine the importance of each target, which provides an indication when scheduling sensors later using the Q-learning framework. Then, the reward function in Q-learning used by MTT-SS encourages the sensors to select a target to track to achieve a balance of resources. Therefore, compared with other scheduling algorithms, MTT-SS can reduce the occurrence of unbalanced allocation of tracking resources for multiple targets and keep the average localization errors in the tracking process to a minimum standard, which is feasible for many practical tracking scenarios.

In the second set of simulations, we evaluate the energy efficiency of the scheduling algorithms by comparing the average residual energy of sensors and the system energy consumption.

**Figure 7:**
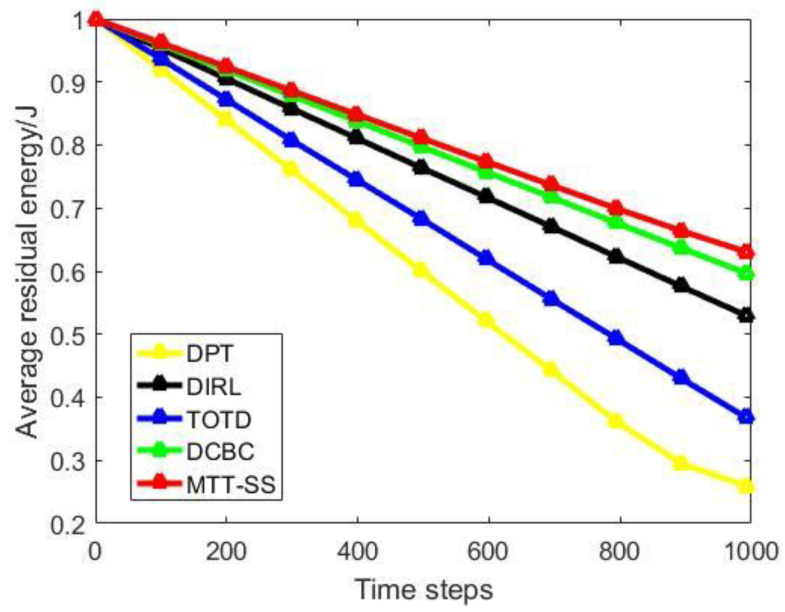
Average residual energy.

**Figure 8:**
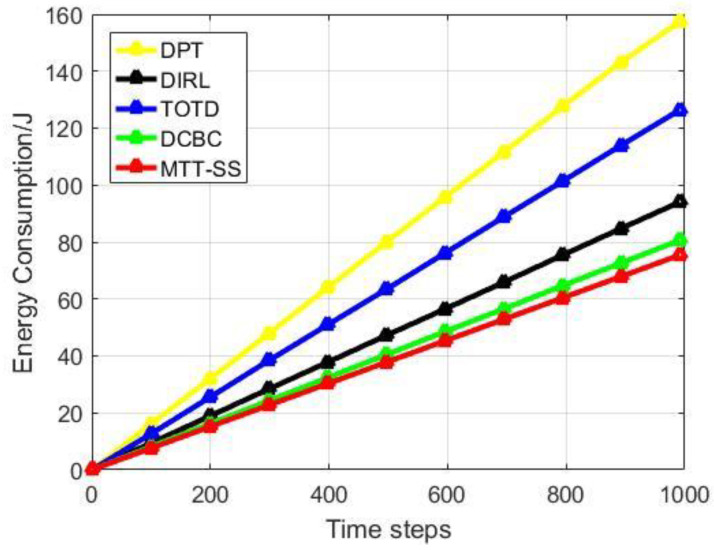
Energy consumption.

Figure 7 provides the comparison of average residual energy based on different sensor-scheduling algorithms. At the 1000-th time step, the average residual energy is 0.257 joules for DPT, 0.526 joules for DIRL, 0.363 joules for TOTD, 0.593 joules for DCBC, and 0.628 joules for MTT-SS. Moreover, the comparison of different sensor-scheduling algorithms in terms of energy consumption is shown in Figure 8. At the 1000-th time step, the energy consumption is 0.257 joules for DPT, 0.526 joules for DIRL, 0.363 joules for TOTD, 0.593 joules for DCBC and, 158.5 joules for MTT-SS. It can be seen that in the proposed MTT-SS, the sensors can use less energy to perform the target-tracking task compared to other algorithms. The reason is that, in the scheduling process of MTT-SS, the setting of the reward function in Equation (14) encourages the sensors to enter the sleep state after all the targets meet the tracking requirements, which reduces the transmission and processing of redundant data. In this way, the transmission and processing of redundant data in the network can be effectively reduced, so the average residual energy of the sensors in the MTT-SS can be kept at a high level. In contrast, DCBC achieves high energy efficiency as well. The reason is that the tracking chain structure of DCBC can ensure that sensors transmit the sensing data to their nearest neighbors for data aggregation, which decentralize the tasks of the cluster head and achieve better energy balance. Moreover, redundant nodes can go to sleep in DCBC in time, so that energy can be further saved. DPT is a traditional dynamic-tracking scheme without an efficient network structure and an effective sensor-scheduling framework, so it performs poorly compared to other methods in terms of energy efficiency. Although DIRL and TOTD have similar sensor-scheduling mechanisms based on reinforcement learning, they lack comprehensive consideration of target attributes, network status, and node capabilities, which makes it difficult for them to allocate resources reasonably in multi-target scenarios. Therefore, they are still inferior to MTT-SS in terms of energy efficiency.

**Figure 9:**
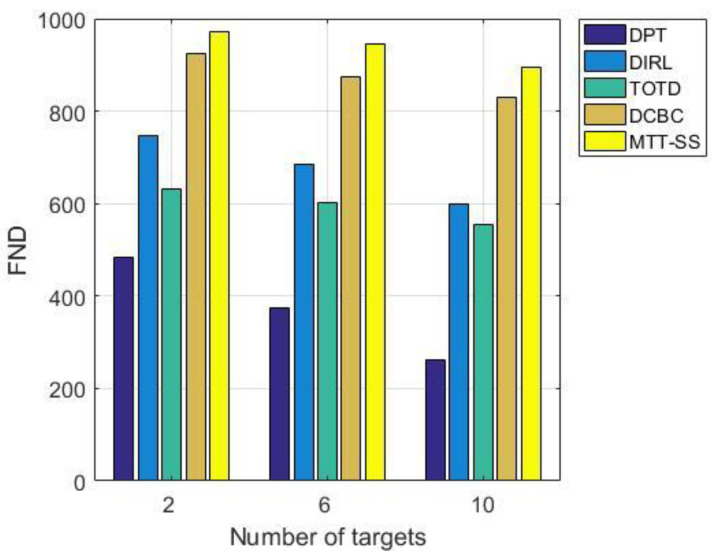
FND time steps vs. number of targets.

Figure 9 shows a comparison of time steps for the first node dead (FND) as the number of targets increases. For target-tracking applications, the tracking performance of the network is greatly reduced once a node in a critical surveillance area dies, thus FND is used to measure the network lifetime. In addition, the time step of FND occurrence can also reflect the load balance of nodes in the network. It can be seen that as the number of targets increases, the time step of FND occurrence for all the algorithms decreases because the sensors undertake more sensing and data processing tasks. When there are six targets in the surveillance area, the average time step for FND is 374 for DPT, 686 for DIRL, 601 for TOTD, 875 for DCBC, and 945 for MTT-SS. MTT-SS gives a higher lifetime than DPT, DIRL, TOTD, and DCBC, which are approximately 153%, 38%, 57%, and 8%, respectively. In DPT, although only sensors near the target need to be activated, there is a lack of scheduling strategies for sensor resources. In order to meet the tracking quality requirements, a large number of sensors need to remain active in DPT, thus causing excessive energy consumption. Both DIRL and TOTD are sensor-scheduling methods based on reinforcement learning. DIRL focuses on the amount of interactive information and energy consumption between sensors when designing the reward function. Additionally, TOTD focuses on the trade-off between network-tracking quality and energy efficiency. Compared with TOTD, DIRL takes more consideration into the agents’ contribution to energy efficiency, so it achieves lower energy consumption and a longer network lifetime. The tracking chain structure of DCBC can improve energy efficiency and balance network energy consumption through dynamic increase and decrease in nodes and the local data fusion mechanism. However, the lack of consideration of resource allocation balance in multi-objective scenarios makes the FND index of DCBC inferior to the proposed MTT-SS. In contrast, the proposed MTT-SS not only considers the requirement for tracking accuracy but also encourages the sensor to sleep when the tracking requirements are met in the reward function. In addition, the consideration of the balance of sensor resource allocation in the exploration–exploitation policy also helps to balance the sensor load. Therefore, MTT-SS has the best performance in terms of network lifetime as defined by FND.

In simulation experiments, we comprehensively evaluate the performance of scheduling algorithms in terms of tracking accuracy and energy efficiency. The results reveal that the proposed sensor-scheduling algorithm can achieve high energy efficiency while ensuring high tracking quality.

## 6. Conclusions

In this paper, we devise and evaluate a sensor-scheduling algorithm for multi-target tracking in WSNs. Firstly, we consider decision indicators, including target type, degree of deviation, node density, and energy level, to construct a target priority function, in which EWM is used to determine the weights of indicators. Then, we propose a Q-learning-based task-allocation mechanism, where sensors are mapped as agents and the tracking status of targets is mapped as environments. Each agent can observe the environment state and select its action using an improved exploration–exploitation policy. By using the proposed sensor-scheduling algorithm, the system can reasonably assign tracking tasks to sensors, thereby improving tracking accuracy and energy efficiency. Simulation results indicate that the proposed algorithm outperforms other algorithms in terms of tracking accuracy and energy efficiency.

## Figures and Tables

**Table 1:** Simulation parameter settings.

Network Scale	100 m × 100 m	$E_{elec}$	50nJ/bit
Sink coordinates	(50 m, 50 m)	$\varepsilon_{fs}$	${10\mathrm{pJ}/\mathrm{bit}/m}^{2}$
Sensing radius	20 m	$\varepsilon_{amp}$	${0.0013\mathrm{pJ}/\mathrm{bit}/m}^{4}$
Communication radius	40 m	Learning rate β	0.8
Size of control packet	8 bytes	Discount factor γ	up and right at an angle of π/6 with the horizontal axis
Size of data packet	256 bytes	Weight coefficient λ	0.4
Time step	1 s	The length of the data packets	600 × 8 bits
Number of sensors	200	Initial energy	0.05 J
Number of targets	(2, 4, 6, 8, 10)	Random motion parameter η	random number ∈ (0, 1)
Initial speed of target	10 m/s	Localization parameter ξ	1.5

**Table 2:** Standard deviation of localization errors.

**Algorithms**	**Standard Deviation** **(m)**
DPT [23]	0.0702
DIRL [24]	0.0562
TOTD [8]	0.0472
DCBC [7]	0.0646
MTT-SS	0.0202

## Data Availability

Not applicable.
